# Attitudes of Australian patients receiving inpatient mental health care towards deprescribing: a cross-sectional survey

**DOI:** 10.1186/s12888-025-06717-3

**Published:** 2025-03-21

**Authors:** Rachel Law, Neeraj Gupta, Aili V Langford, Emily Reeve

**Affiliations:** 1 Rural and Remote Mental Health Services, Older Persons Mental Health Specialised Services, Adelaide, SA, South Australia Australia; 2 Northern Metropolitan Child and Adolescent Mental Health Service (CAMHS), SA Health, Adelaide, South Australia Australia; 3 University of Adelaide, Adelaide, South Australia Australia; 4 Sydney Pharmacy School, Faculty of Medicine and Health, The University of Sydney, Camperdown, Australia; 5 Centre for Medicine Use and Safety, Faculty of Pharmacy and Pharmaceutica l Sciences, Monash University, 381 Royal Parade, Parkville, VIC 3052 Australia; 6 Quality Use of Medicines and Pharmacy Research Centre, Clinical and Health Sciences, University of South Australia, Adelaide, SA Australia

**Keywords:** Deprescribing, Patient attitudes, Mental health

## Abstract

**Background:**

Psychotropic polypharmacy is common, increasing, and associated with higher risks of adverse effects, hospitalisations and mortality. This study aimed to explore the attitudes and beliefs of people receiving inpatient mental health care toward deprescribing (*discontinuing a medication when the current or potential risk outweighs the current or potential benefit*) and determine any patient characteristics associated with these attitudes and beliefs.

**Methods:**

A cross-sectional survey of patients admitted to two open acute psychiatric inpatient units was conducted over a 6-month period in the Australian metropolitan city of Adelaide. Individuals were eligible to participate regardless of their reason for admission, if they were at least 18 years old and able to converse, read and write in English, and provide informed consent. Participant characteristics and responses to the validated revised Patients’ Attitudes Towards Deprescribing (rPATD) questionnaire were collected. The rPATD includes questions grouped into four factors: (i) perceived burden of medications, (ii) involvement in medication management, (iii) belief in appropriateness of medications, and (iv) concerns about stopping, plus two global questions. Participants were encouraged to think about medications that they use for their mental health conditions when completing the questionnaire.

**Results:**

One hundred participants were recruited, with a mean age of 41.6 (SD = 13.7). 65% of participants agreed that they would be willing to stop one or more of their psychotropic medications if their doctor said it was possible. In a binary logistic regression model, willingness to have a medication deprescribed was mostly strongly predicted by Involvement factor score (odds ratio [OR] = 5.92, 95% confidence interval [CI] = 2.10-15-16.70, *p* < 0.001).

**Conclusions:**

A majority of participants were open to having one or more medication deprescribed. When medically justified, mental health professionals should feel comfortable initiating conversations about deprescribing to understand patient attitudes and preferences, fostering shared decision-making for psychotropic medication management.

**Clinical trial number:**

Not applicable.

**Supplementary Information:**

The online version contains supplementary material available at 10.1186/s12888-025-06717-3.

## Background

The World Health Organisation and the United Nations have asserted that it is a human right for patients to be aware of the ability to cease treatments such as medications, and that there should be support available to them to do this [1]. *Deprescribing* is the systematic process of identifying and discontinuing a medication when the current or potential risk of continued therapy outweighs the current or potential benefit, taking into consideration an individual’s functional status, care goals, values and preferences [2]. To date, deprescribing research has focussed predominantly on older adults [3] and certain high-risk medications, including benzodiazepines and opioid analgesics [4, 5]. There has been limited research examining perspectives of deprescribing in people living with mental illness, despite it being of particular relevance for this cohort [6].

Psychotropic polypharmacy (*the use of two or more psychotropic medications concurrently* [7]) is common, rising, and associated with increased adverse effects (e.g. metabolic syndrome, movement disorders), hospitalisations and mortality [6, 8–10]. For example, an Australian cohort study found that the prevalence of psychotropic polypharmacy in adults was 11.1% in 2006, increasing to 12.0% in 2015 [11]. An increase in the prevalence of class polypharmacy (*2 or more drugs from within the same drug class*) was observed, rising from 5.9 to 7.3% for antipsychotics, 2.1–3.7% for antidepressants and 2.9–4.3% for benzodiazepines [11]. Similarly, the concurrent use of two or more antipsychotics specifically for conditions such as treatment-resistant schizophrenia is common practice. A systematic review found that approximately a third of people with schizophrenia spectrum disorders received more than one antipsychotic medication [12]. Use of two or more antipsychotics is associated with increased risk of relapse, hospitalisation, worsened functioning, mortality, and adverse effects [12]. There is limited evidence to support a benefit of using more than one antipsychotic [13, 14], and several studies have found that the majority of people on antipsychotic polypharmacy were able to tolerate conversion to monotherapy [15–17], and this may even result in a benefit [18]. For this reason, The Royal Australian and New Zealand College of Psychiatrists (RANZCP) recommend that if a patient is receiving two or more antipsychotic medicines, the medication regime should be regularly reviewed and simplified if possible [19]. Research suggest both a need and an opportunity to enhance medication appropriateness and consider deprescribing of psychotropic medications to reduce polypharmacy and associated harms for individuals living with mental illness.

Even in the absence of psychotropic polypharmacy, medication use in people living with mental illness can have changing benefits and harms over time. In the treatment of schizophrenia, antipsychotics can acutely lead to an improvement in total symptoms and positive, negative and depressive symptoms [20]. This can extend to improved quality of life and social functioning [20]. But response to antipsychotics is highly heterogeneous, and long term use can lead to multiple serious adverse effects, such as metabolic and movement disorders [20–23]. Therefore, not every patient derives more benefit than harm or requires long-term treatment [24] and the suitability of long-term use needs to be determined on an individual basis. Chronic use of other psychotropic medicines, specifically benzodiazepines, may also confer a greater risk of harm in patients with psychiatric disorders and can contribute to cognitive impairment, behavioural disinhibition, paradoxical agitation and exacerbation of psychotic symptoms [25–27]. Moreover, a 70% increased risk of death including suicide has been observed when benzodiazepines are prescribed at high doses in patients with schizophrenia [28]. Recognising the significant potential harms of psychotropic medications, coupled with the knowledge that a patients’ understanding of their illness and coping strategies may evolve over time, regular re-assessment of the appropriateness of ongoing therapies and consideration of the suitability of deprescribing is warranted.

There is emerging evidence that deprescribing high risk medications is feasible and safe in people living with mental illness [29–32]. However, deprescribing may also be deemed particularly complex in this population due to anticipated resistance from patients, families and healthcare professionals. Fears may include that deprescribing will lead to a relapse of the condition or worse patient outcomes [33]. However, shared decision making may act as a tool for health professionals and people living with mental illness to discuss and address such fears, empower patients to be actively involved in the management of their condition, encourage help-seeking behaviours and reduce stigma [34].

To support shared-decision making, we must first elicit the perspectives of the general adult mental health population toward deprescribing [35, 36]. This study aimed to quantitatively explore the attitudes and beliefs of a mental health population toward deprescribing and to identify what patient characteristics, if any, were associated with these attitudes and beliefs.

## Methods

Ethics approval was obtained from Central Adelaide Local Health Network Human Research Ethics Committee (CALHN HREC) and Barossa Hills Fleurieu Local Health Network (BHFLHN HREC). The manuscript is reported in accordance with the STROBE cross sectional reporting guidelines (Additional File 1) [37].

### Study design and setting

In the psychiatric setting, hospitalisation provides an impetus and an opportunity to review medications as part of treatment optimisation to address the reason for admission. Hospitalisation may be a suitable environment for deprescribing in people with mental health conditions as it provides access to a multidisciplinary team to support decision-making and an opportunity for multi-day discussions and short-term close monitoring. Alternatively, it may provide an opportunity to start a discussion about deprescribing (as it may not be appropriate to deprescribe while they are an inpatient) that could lead to recommendations to consider deprescribing after the patient has been discharged.

This cross-sectional study was conducted on two open acute psychiatric inpatient units based in the metropolitan Australian city of Adelaide, over a 6-month study period. Open units are ones in which the patients have freedom of movement, unlike secure or locked facilities. One unit primarily catered to metropolitan-based patients, while the other catered to rural patients.

All individuals admitted to the two acute psychiatric units, regardless of their reason for admission, were eligible to participate, ensuring representation of a broad spectrum of conditions. Individuals were invited to participate in the study if they were at least 18 years old, able to converse, read and write in English and able to provide informed consent. Participants were excluded if they were under the influence of illicit substances, cognitively impaired, too unwell to participate as judged by their treating team or did not consent to participate.

Data collection occurred from the 15th of December 2022 to the 20th of June 2023. Written informed consent was obtained prior to data collection.

### Data collection

Participant attitudes and perceptions about medication use were captured using the revised Patients’ Attitudes Towards Deprescribing (rPATD) questionnaire. The rPATD is a validated questionnaire used to assess patient and/or carer attitudes towards deprescribing. It has a moderate internal consistency (Cronbach’s α > 0.65), and test-retest reliability (gamma values between 0.57and 0.89, *p* < 0.001) [38]. The rPATD questionnaire has been used in multiple research studies internationally in a variety of different populations including older persons in the general medical hospital and community settings and in older adults with mental health illnesses [39–41].

The rPATD uses a 5-point Likert scale from strongly agree to strongly disagree to capture responses to statements. The questionnaire consists of 22 questions. They are divided into 4 factor scores namely, (i) appropriateness score, (ii) involvement score, (iii) burden score and (iv) concerns about stopping score, as well as two global questions (“If my doctor said it was possible, I would be willing to stop one or more of my regular medicines” and “Overall, I am satisfied with my current medicines”). No changes were made to the wording of the questions, however, at the beginning of the questionnaire, participants were advised to consider the medications that they use for their mental health conditions when answering the questions. For the questions that ask about their ‘doctor’ they were directed to think of the doctor that prescribes most (if not all) of their mental health medications. A member of the treating team (the medical team responsible for the patients care while in hospital) conducted an eligibility assessment to determine if a patient could be invited to participate. For pragmatic reasons patients were approached at any point during their admission. After obtaining written consent, the primary investigator (RL; a psychiatry registrar) provided the study questionnaire to each participant, who could elect to self-complete the questionnaire with no assistance, with some assistance from the researcher, or ask the researcher to administer the questionnaire (i.e. read out the questions and note down their answers). Participants were informed that the investigator was not part of their treating team and that their responses would be kept confidential. Participants were not compensated for participation. Paper versions of the rPATD survey and a participant data collection sheet were used to collect the data. Data on demographics, clinical characteristics and medication use were captured during the interview, supplemented by participants’ medical records.

A sample size of 51 was calculated based on an estimated agreement to the willingness to deprescribe question of 80%, allowing 10% precision and a 95% confidence interval [42]. We aimed to recruit 100 participants to account for dropouts and missing data and were driven by best practice recommendations to have a minimum of 100 participants for surveys.

### Statistical analysis

Descriptive statistics were used to summarise participant characteristics and the results of the rPATD. Factor scores of between 1 and 5 were created by summing the responses to each statement in the factor then dividing them by the number of items in the factor. The direction of the score was created for higher scores to illustrate a higher perception of burden, appropriateness, concerns about stopping medications and involvement (including perceived knowledge of their medications and involvement in making decisions) [38]. That is, for the burden, concerns about stopping and involvement factors responses were scored from 1 for strongly disagree to 5 for strongly agree. For the appropriateness factor, these scores were reversed.

A gamma rank correlation was used to examine the relationship between factor scores and willingness to deprescribe and comfort with medications (global questions). A binominal logistic regression analysis was conducted to determine predictors of agreement to the question: “*I would be willing to stop one or more of my medicines if my doctor said it was possible*” (a global question of the rPATD; dependent variable). For the purpose of regression analysis, this question was converted to a binary outcome of agree vs. not agree. Responses of “strongly agree” or “agree” were grouped as agree and “strongly disagree”, “disagree,” or “unsure” were grouped as not agree. Participant characteristics (gender, age, psychotropic polypharmacy, ward) and rPATD factor scores were considered as independent variables. For this analysis, the factor scores were converted to a binary variable; participants were considered to have a ‘high’ factor score if they scored equal to or greater than the median of the population, with the remainder being classified as having a ‘low’ factor score. These independent variables were decided a priori based on previous studies with the rPATD and clinical experience of the research team. All analyses were conducted using IBM SPSS Statistics (Version 28).

## Results

### Recruitment and participant characteristics

During the study recruitment period, there were 319 and 179 discharges from the metropolitan inpatient unit and the rural and remote inpatient unit respectively. Of 498 potential participants, 124 were deemed eligible by the treating team to be approached for the study. One hundred (80.6%​) participants consented to take part in the study. Participant characteristics are presented in Table 1.

Participants had a mean age of 41.6 (standard deviation: 13.7) years. Approximately half (51%) of participants were male, 85% were of Caucasian ethnicity and 12% identified as Aboriginal or Torres Strait Islander.

A third (38%) of participants had a primary diagnosis of psychotic illness, a third (35%) had a mood disorder, and 3% had anxiety. Overall, 62% of participants were taking two or more regular psychotropic medications and 7% were taking five or more non-psychotropic medications. Most (83%) participants were responsible for managing their own medications, 13% were managed by a care coordinator and 4% were managed by family or friends. The majority (86%) of participants wanted to have their medications reviewed by a doctor or pharmacist.

**Table 1:** Participant characteristics (*n* = 100)

PARTICIPANT CHARACTERISTICS	*n* = 100
**Age**,** mean (SD)**	41.6 (13.7)
**Male**,** (%)**	51
**Ethnicity**,** (%)**	
Caucasian	85
Aboriginal and/or Torres Strait Islander	12
Other	3
**Marital status**,** (%)**	
Married/de facto	26
Single	68
Other	6
**English as first language**,** (%)**	97
**Born in Australia**,** (%)**	95
**Highest level of education completed**,** (%)**	
Less than year 12	13
High school	25
Trade/apprenticeship	29
Certificate/diploma	20
Bachelor	12
Postgraduate	1
**Ward**,** (%)**	
Rural and remote inpatient unit	51
Metropolitan inpatient unit	49
**Primary diagnosis of admission**,** (%)**	
Psychosis	38
Mood disorder	35
Anxiety	3
Other	24
**Co-morbid medical issues**,** (%)**^**$**^	
Metabolic medical issues	20
Non-metabolic medical issues	45
**Psychotropic medication use**,** (%)**^**%**^	
Antidepressant	52
Antipsychotic	72
Benzodiazepine	16
Mood stabiliser	27
**Non-psychotropic medication use**,** (%)**^**%, §**^	
Antihypertensive	10
Antidiabetic	11
Anticholesterol	12
Vitamins	17
Analgesic	7
Antacid	8
Laxative	2
**No. of regular psychotropic medications**,** (%)**^**^**^	
1	38
2	39
3	15
4	4
5	3
6	1
**No. of regular non-psychotropic medications**,** (%)**^**^**^	
< 2	72
2–4	21
5–6	4
7–10	3
**Responsibility of medication management at home**,** (%)**	
Self	83
Family/friends	4
Care coordinator/support worker	13
**Interested in having a medication review**,** (%)’**	
Yes	86
No	7
Unsure	7

^$^participants can have both metabolic and non-metabolic medical issues, or neither

^%^Percentage (%) could add up to more than 100 as participants could be on more than one of these classes

^§^regular prescription and non-prescription medications were included

^regular medications are medications charted and taken on a scheduled basis at the time of survey completion

^’^ Participants were asked: “Would you like to have all your medications reviewed by a doctor or pharmacist?”

### Beliefs about mental health medications and attitudes towards deprescribing

**Fig. 1:**
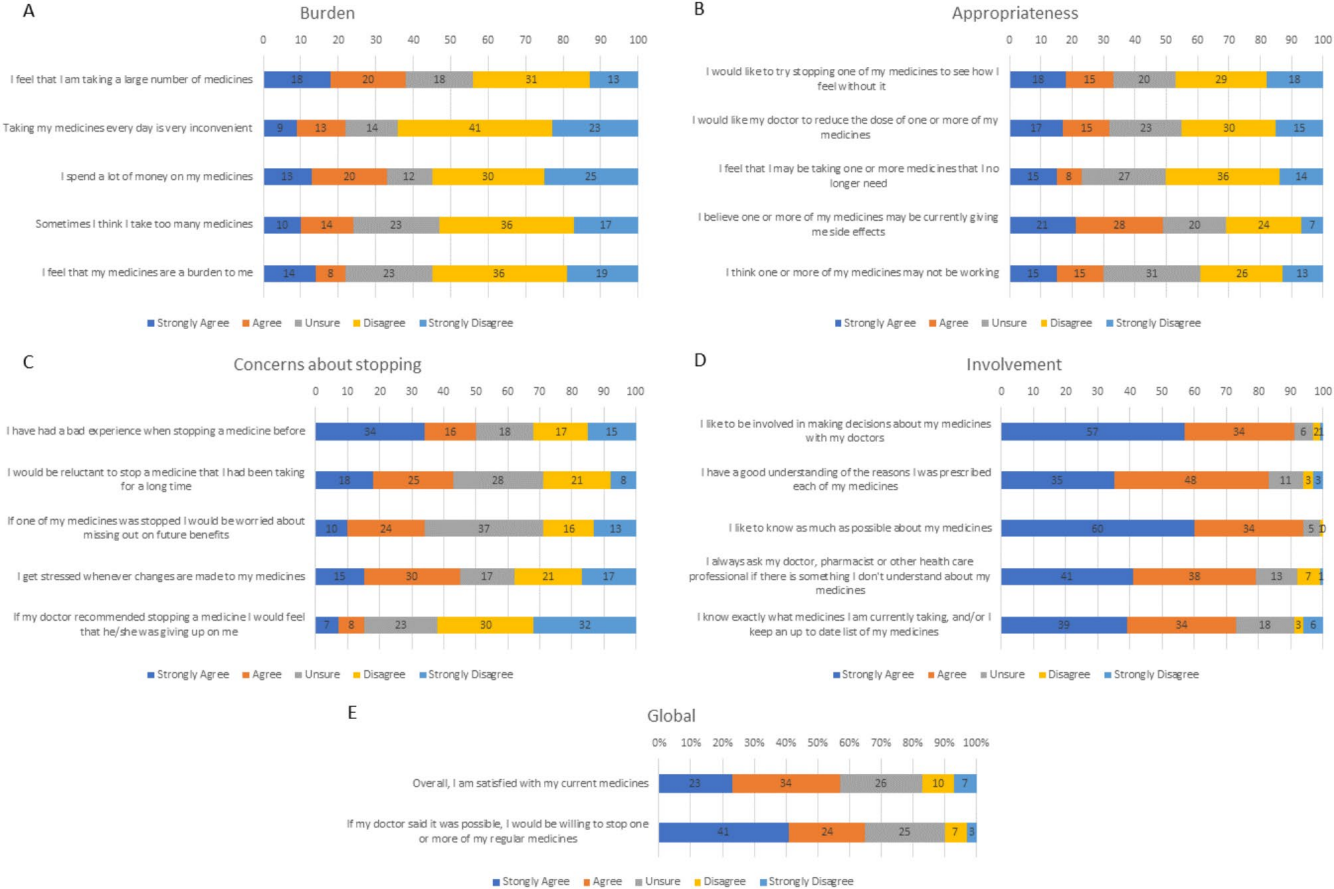
Responses to the rPATD questions (*n* = 100) (**A**) Burden factor questions. (**B**) Appropriateness factor questions. (**C**) Concerns about Stopping factor questions. (**D**) Involvement factor questions. (**E**) Global questions

Almost half (43%) of the participants were dissatisfied or had mixed feelings about their medications. 65% (65%) agreed that they would be willing to stop one or more of their psychotropic medications if their doctor said it was possible. Most of the participants (91%) liked to be involved in decision making about their medications and the majority (94%) were interested in knowing as much as they could about their medications. 83% of participants reported good understanding of the rationale for their medications. Approximately one quarter (22%) felt burdened by their medication and half (49%) believed that one or more of their medicines may be currently giving them side effects. However, almost half (45%) of the participants reported experiencing stress with any alterations to their medication (Fig. 1). Factor scores are presented in Table 2.

**Table 2:** Factor scores

Factor scores	Median, *n* = 100	IQR, 1st quarter − 3rd quarter,
**Burden**: Perceived burden of medications	2.8	2.0–3.2
**Appropriateness**: Belief in appropriateness of medications	3.2	2.4–3.8
**Concerns about stopping**: concerns about stopping medications	3.0	2.2–3.75
**Involvement**: involvement in medication management	4.2	4.0–4.8

Possible score range: 1–5. Higher score indicates greater perceived burden of medications, belief in appropriateness of medications, concerns about stopping, and involvement with medications

Questions in the burden, concerns about stopping and involvement factors were scored: strongly agree = 5, agree = 4, unsure = 3, disagree = 2, strongly disagree = 1. Questions in the appropriateness factor were scored: strongly agree = 1, agree = 2, unsure = 3, disagree = 4, strongly disagree = 5

Abbreviation: IQR, interquartile range

### Association between participant characteristics and willingness to have medications deprescribed

Table 3 describes the correlation between the factor scores and the global questions. The burden and the belief in appropriateness factors had the strongest correlation of those examined (negative correlation; G= -0.473, *P* = < 0.001). Lower concerns about stopping (G=-0.250; *p* = 0.018), greater involvement (G = 0.303, *p* = 0.003), increased burden (G = 0.253, *P* = 0.014) and decreased belief in appropriateness (G=-0.383 *P* = < 0.001) were associated with being more willing to cease a mental health medication. Higher involvement (G = 0.47; *p* < 0.001), increased belief in appropriateness (G = 0.332 *P* = 0.002) and decreased burden (G=-0.325; *p* < 0.001) were associated with increased overall satisfaction with current mental health medications.

**Table 3:** Correlation between factor scores and global questions

	Appropriateness Gamma value, *p* value	Concerns Gamma value, *p* value	Involvement Gamma value, *p* value	If my doctor said it was possible I would be willing to stop one or more of my regular medicines Gamma value, *p* value	Overall, I am satisfied with my current medicines Gamma value, *p* value
**Burden**	**-0.473** *P* ** = < 0.001**	0.126 *P* = 0.122	-0.060 *P* = 0.474	**0.253** *P* ** = 0.014**	**-0.325** *P* ** = < 0.001**
**Appropriateness**		-0.022 *P* = 0.801	0.143 *P* = 0.092	**-0.383** *P* ** < 0.001**	**0.332** *P* ** = 0.002**
**Concerns**			0.047 *P* = 0.566	**-0.250** *P* ** = 0.018**	0.171 *P* = 0.081
**Involvement**				**0.303** *P* ** = 0.003**	**0.47** *P* ** < 0.001**
**If my doctor said it was possible I would be willing to stop one or more of my regular medicines**					0.037 *P* = 0.763

Significant associations shown in bold

Gamma value, *p* value (Gamma rank correlation)– a positive gamma value indicates that as perception of burden, appropriateness of medications, concerns about stopping and involvement increases, agreement with the statement increases. E.g. greater reported involvement was associated with greater reported willingness to have a medication withdrawn. For negative gamma values, this meant that they were more likely to disagree. Ordinal data was used for these analysis (the factor score or the 5-point Likert response for the global questions)

All four factor scores and selected participant characteristics were included in a binary logistic regression model. In this model high involvement (odds ratio [OR] = 5.92, 95% confidence interval [CI] = 2.10-15-16.70, *p* < 0.001) and low appropriateness factor scores (odds ratio [OR] = 0.18, 95% confidence interval [CI] = 0.52–0.60 *p* < 0.006) enhanced the probability of willingness to have a medication deprescribed (Table 4).

**Table 4:** Predictors of willingness to have a medication deprescribed (binary logistic regression analysis)

Independent variable	aOR	95% CI
High Burden score (≥ 2.8)	1.10	0.35–3.46
**High Appropriateness score (≥3.2)**	**0.18**	**0.52–0.60**
High Concerns about stopping score (≥ 3.0)	0.41	0.15–1.11
**High involvement score (≥4.2)**	**5.92**	**2.10–16.70**
Male gender	2.04	0.77–5.39
Age	1.00	0.97–1.04
Psychotropic polypharmacy*	0.98	0.34–2.89
Metropolitan ward^#^	1.67	0.64–4.34

The logistic regression model was significant (*p* < 0.001), had a good model fit (Hosmer-Lemeshow test *p* = 0.87), explains 30.6% of the variance (Nagelkerke R^2^) and correctly predicts 75% of the results

Abbreviations: aOR, adjusted odd ratio; CI, confidence interval

Bolded values are significant

*Use of two or more psychotropic medications versus use of one psychotropic medication

^#^ Ward that primarily services people living in metropolitan areas versus a ward that primarily services people living in rural or remote areas

## Discussion

Existing deprescribing literature predominantly focuses on older adults with multimorbidity and polypharmacy. There is limited primary research to support deprescribing in the field of psychiatry. To our knowledge, this is the first quantitative investigation of attitudes towards deprescribing in the adult (aged 18 years and older) inpatient population living with mental illness.

In this study, two thirds of participants (65%) were willing to have one or more of their medications deprescribed if their doctor said it was possible. Although this represents a large proportion of the cohort, results are lower than those reported in previous studies of different populations. A meta-analysis of 40 studies employing the rPATD found that 84% of participants were willing to have a medication deprescribed if their doctor said it was possible [41]. Similarly, two studies of older patients with psychiatric disorders reported that 92% and 77% of participants respectively, were willing to stop a medication on their physician’s advice [39, 40]. The lower values obtained in this study could be attributable to multiple factors. Firstly, the patient population in this study was substantially younger than previous studies and a higher mean age has been associated with a higher willingness to stop medications [43, 44]. Secondly, symptom-modifying medications have been reported to be more difficult to deprescribe than risk-modifying medications [45]. As participants were asked to consider their mental health medications when completing the questionnaire, it is likely that eligible medications were predominantly symptom-modifying. Lastly, study participants were recovering from a significant relapse requiring inpatient treatment at the time of survey administration. Hence, they may have had more recent insight into the role of medications in their stability. There may also have been influence from psychoeducation by inpatient clinicians regarding the importance of medication adherence in reducing the risk of relapse and maintaining response to treatment. The factor scores for involvement and burden in this population are similar to previous studies with older adults in the community and in hospital in Australia [46, 47]. The appropriateness and concerns about stopping scores may have been slightly higher in our population [46, 47]. Given the limited sample size in this and one of the previous studies, the interpretation of this is unclear. However, it may reflect the high rate of anxiety in this population (increasing concerns in general) and the proximity to an acute relapse (i.e. requiring hospitalisation). In previous studies using the rPATD, most studies report high satisfaction with medications (> 80%) while in this study it was only 65%. This is similar to the rates of favourable attitudes towards treatment in people living with mental illnesses [48, 49]. This population has high rates of nonadherence, and there is a need for more education for people living with mental illnesses, especially about their medications, and efforts to reduce the stigma of diagnoses and treatment [50–52].

Factors score analysis demonstrated a negative correlation between appropriateness score and willingness to have a medication deprescribed. People with low appropriateness scores could have concerns about previous, ongoing or potential adverse effects, stigma associated with psychotropic medications or fears about dependence [53, 54]. Almost half (49%) of the study participants believed that one or more of their medicines may be causing adverse effects, a figure higher than what has previously been reported in other populations [41]. This may reflect the rapid titration of psychotropic medications to therapeutic doses in the inpatient setting due to the acuity of the population [55], as adverse effects are commonly dose-dependent [56]. However, such adverse effects may contribute to intentional medication nonadherence, a phenomenon of particular pertinence in the field of psychiatry. Ambivalence about diagnosis and treatment is common due to the direct effects of the illness on insight and judgement, as well as treatment-related stigma. Patients who experience adverse effects and view their medications as less appropriate, may be more likely to stop them, which may lead to symptom relapse, illness exacerbation, re-hospitalisation, and an increased risk of suicide [53, 54]. A purported benefit of deprescribing inappropriate medications is that it may help to improve adherence to necessary medications [57], however, caution must be exercised when determining the suitability of deprescribing based on adverse effects alone. Instead, clinicians should facilitate regular and open discussions about the benefits and harms of both medication continuation and discontinuation to ensure appropriate medication use. Our binary logistic regression model found that the strongest predictor of willingness to have a medication deprescribed was a high involvement score. Increased patient participation in decision making may therefore not only facilitate deprescribing but also increase treatment adherence as mental health patients have indicated a strong preference for involvement in decision making about their care [58, 59]. This also supports current knowledge of the importance of patient centred care and empowerment in the mental health field [60]. Our findings indicate that increasing shared decision making (i.e. involvement) and educating people with mental illnesses about why their medications may be inappropriate may increase willingness to deprescribe. Discussion of burden and concerns about stopping (while not associated in this study) also represents best practice as this is important to achieve shared decision making.

We did not find an association between psychotropic polypharmacy and willingness to deprescribe. Previous studies have shown varied associations (or lack thereof) with number of medications and attitudes [41]. Additionally, there has been no association between willingness and use of inappropriate medications, highlighting that there may be limited consumer knowledge about medication appropriateness. We only included a small number of participant characteristics in our binary logistic regression (gender, age, psychotropic polypharmacy and ward) and so further research which includes additional characteristics, such as medication adherence, class polypharmacy, diagnoses, reason for admission (e.g. if medication related) and the influence of different medications is needed. Additionally, whether willingness to deprescribe differs at different times during a patient care journey (e.g. when they are not in hospital) needs to be explored.

Although the study findings demonstrate willingness of mental health patients to deprescribe, medication reduction or cessation will inevitably not be appropriate for all patients. Individuals with a history of suicide or homicide attempts, multiple recent hospitalisations, severe relapses after previous deprescribing attempts, or poor social support may be unsuitable candidates [6]. Even when deprescribing is deemed appropriate, there may be additional challenges and considerations, including a risk of precipitating relapse, withdrawal symptoms, and reinforcing stigma associated with medication use [35]. Gupta et al. have proposed a process for deprescribing in psychiatry, addressing some of these challenges [61]. Additionally, new evidence-based tools and resources such as the Maudsley Deprescribing Guidelines [62] have been released to support mental healthcare professionals and patients to achieve safe and appropriate reduction or cessation of psychiatric medications. However, more research is required to identify patients for whom deprescribing might be most appropriate and successful.

Given the nature of this quantitative questionnaire, an in-depth exploration of the reasons for the observed associations were unable to be captured. Future qualitative studies such as in-depth interviews with mental health populations and other stakeholders (e.g. caregivers and clinicians) from various settings will be helpful to ascertain further information about patients attitudes toward deprescribing. Research should also focus on attitudes of other stakeholders such as psychiatrists, general practitioners, mental health clinicians and caregivers [63]. Whilst research on the barriers and facilitators to deprescribing has been conducted in the field of child and adolescent psychiatry [64, 65], it is not known whether the same or unique barriers exist in the adult population.

### Study limitations

There are several potential limitations to this study. Although the rPATD is a tool that has been used in multiple research studies, both locally and internationally, within the general medical setting, it has not been previously employed in the adult inpatient mental health population. Whilst participants were encouraged to consider their mental health medications when completing the questionnaire, we cannot be confident that the findings represent participants attitudes specifically toward their mental health medicines only. In addition, the responses to study questionnaire may be susceptible to social desirability bias (i.e. giving a response that is perceived to be the ‘correct’ or right way to answer the question), especially if the researchers were seen as part of the healthcare team providing their care in hospital. We attempted to reduce this bias by being explicit about the confidentiality of the responses, informing participants that the investigator was not involved in their care and allowing participants to self-administer the questionnaire whenever practical. Additionally, we included any person admitted to the acute psychiatric unit, regardless of their reason for admission. The majority had a primary diagnosis of psychosis or mood disorders, however, approximately a quarter of participants had an ‘other’ diagnosis. Based on our sample size we were not able to do any sub-group analyses to determine whether there were differences in attitudes between people with different mental health conditions. Participants were approached to complete the survey at different points during their stay, and it is possible that attitudes may vary during an acute admission period.

Approximately three quarters of potentially eligible participants were deemed unsuitable to be approached for participation in the study by their treating team. For confidentiality reasons, we do not know the reasons why they were excluded. The participants in this study were similar in age and gender breakdown to previous studies of this population in Australia. However, there was a lower proportion of people from cultural and linguistically diverse (CALD) backgrounds who may have different beliefs or health literacy levels when compared to the examined population [66]. Similarly, although this study was conducted in psychiatric inpatient units, it did not include patients who were treated involuntarily, and it is possible that the participants do not represent mental health patients in the community. Therefore, these results may not be generalisable to other distinct patient cohorts. We have no information about patients who chose not to participate and whether their participation could have significantly altered our study findings.

## Conclusions

Two thirds of adult patients receiving inpatient mental health care were willing to have one or more of their medications deprescribed. As such, mental health professionals should feel confident to openly broach the topic of deprescribing with their patients, without being overly concerned about encountering resistance. Encouraging patient involvement in medication decisions and addressing concerns about stopping medications are likely to contribute to enhancing shared decision making in this process.

## Electronic supplementary material

Below is the link to the electronic supplementary material.

Supplementary Material 1

## Data Availability

The data generated during and/or analysed in this study are not publicly available due to privacy and ethical restrictions. However, de-identified data may be available from the corresponding author on reasonable request.

## Abbreviations

**CALD**: Cultural and linguistically diverse
**rPATD**: Revised patients’ attitudes towards deprescribing
**RANZCP**: Royal australian and new zealand college of psychiatrists
